# HHV-6A/6B Infection of NK Cells Modulates the Expression of miRNAs and Transcription Factors Potentially Associated to Impaired NK Activity

**DOI:** 10.3389/fmicb.2017.02143

**Published:** 2017-10-31

**Authors:** Roberta Rizzo, Irene Soffritti, Maria D’Accolti, Daria Bortolotti, Dario Di Luca, Elisabetta Caselli

**Affiliations:** Section of Microbiology and Medical Genetics, Department of Medical Sciences, University of Ferrara, Ferrara, Italy

**Keywords:** HHV-6, miRNA, transcription factors, natural killer cells, virus infection

## Abstract

Natural killer (NK) cells have a critical role in controlling virus infections, and viruses have evolved several mechanisms to escape NK cell functions. In particular, Human herpesvirus 6 (HHV-6) is associated with diseases characterized by immune dysregulation and has been reported to infect NK cells. We recently found that HHV-6 *in vitro* infection of human thyroid follicular epithelial cells and T-lymphocytes modulates several miRNAs associated with alterations in immune response. Since miRNAs are key regulators of many immune pathways, including NK cell functions, we aimed to study the impact of HHV-6A and -6B *in vitro* infection on the intracellular mediators correlated to NK cell function. To this purpose, a human NK cell line (NK-92) was infected *in vitro* with HHV-6A or 6B and analyzed for alterations in the expression of miRNAs and transcription factors. The results showed that both viruses establish lytic replication in NK-92 cells, as shown by the presence of viral DNA, expression of lytic transcripts and antigens, and by the induction of an evident cytopathic effect. Notably, both viruses, although with species-specific differences, induced significant modifications in miRNA expression of miRNAs known for their role in NK cell development, maturation and effector functions (miR-146, miR-155, miR-181, miR-223), and on at least 13 miRNAs with recognized role in inflammation and autoimmunity. Also the expression of transcription factors was significantly modified by HHV-6A/6B infection, with an early increase of ATF3, JUN and FOXA2 by both species, whereas HHV-6A specifically induced a 15-fold decrease of POU2AF1, and HHV-6B an increase of FOXO1 and a decrease of ESR1. Overall, our data show that HHV-6A and -6B infections have a remarkable effect on the expression of miRNAs and transcription factors, which might be important in the induction of NK cell function impairment, virus escape strategies and related pathologies.

## Introduction

Natural killer (NK) cells belong to the innate immune system and are essential effector cells in the control of virus infections (Cooper et al., 2001). Their activity during antiviral response is crucial to control initial virus replication, by killing virus-infected cells prior to the development of adaptive/specific immunity, but they also act as essential regulators of the adaptive immunity (Vivier et al., 2008). Their relevance is supported by the observation that individuals with defects in the NK cell component of the innate immunity are more susceptible to virus infection (Orange, 2002), including herpesviruses (Fleisher et al., 1982; Biron et al., 1989), and consequently more prone to develop symptomatic disease following infection. On the other hand, the importance of NK cell activity during virus infections is reflected by the many mechanisms acquired by viruses to evade NK cell-mediated immune responses (Vossen et al., 2002; Arens, 2012). In particular, human herpesvirus 6A and 6B (HHV-6A and 6B), as all viruses belonging to the *Herpesviridae* family, have developed several mechanisms to control and inactivate the immune response in order to establish a lifelong infection in their hosts.

HHV-6A and 6B are members of the *Roseolovirus* group of the β *herpesvirinae* subfamily and, although they share high sequence homology, are classified as distinct species. In fact, they show important differences in biologic properties, epidemiology, and disease association (Ablashi et al., 2014). HHV-6B infects humans in early childhood and is responsible of *Exanthem subitum* (Yamanishi et al., 1988), while primary infection with HHV-6A still has to be clearly identified. Both HHV-6A and -6B establish a latent infection in the host following resolution of primary infection. Reactivations in the adult have been associated to the development of multiple symptomatic diseases often characterized by immune dysregulation (multiple sclerosis, Sjögren’s syndrome, autoimmune thyroiditis, and others) (Caselli and Di Luca, 2007). Both viruses are considered lymphotropic, showing an elective tropism for CD4+ T-lymphocytes and being able to infect several different cell types of the immune system, including NK cells (Lusso et al., 1993; Caselli and Di Luca, 2007).

Interestingly, *in vivo* and *in vitro* evidences indicate that HHV-6A/6B interfere with the immune system of the infected host in several ways (Lusso, 2006; Dagna et al., 2013). They can modulate surface antigens important for T-cell activation, such as human leukocyte antigen (HLA) class I molecule expression in dendritic cells (Hirata et al., 2001); they also can affect cytokine and chemokine productions, including selective suppression of IL-12, affecting the generation of effective cellular immune responses (Smith et al., 2003; Dagna et al., 2013). Furthermore, we recently observed that HHV-6A infection induces the expression of the tolerogenic non-classical class I HLA-G molecule in primary human mesothelial cells, leading to impairment of NK cell recognition and killing of infected cells (Caselli et al., 2015). With reference to the NK cell component of the immune response, HHV-6A was reported to establish a productive infection in CD3-negative NK cell clones, leading to the *de novo* expression of CD4 on the NK cell surface (Lusso et al., 1993), and HHV-6B was recently shown to induce down-modulation of the activating NKG2D ligand in infected cells (Schmiedel et al., 2016).

Notably, it has been recently reported that NK cells may be directly involved in the onset and progression of autoimmune diseases, through their potential autoreactivity or through their interaction with the other immune cells (Schleinitz et al., 2010; Poggi and Zocchi, 2014), thus supporting the hypothesis of a correlation between HHV-6A/6B infection, NK cell function and autoimmunity.

On the other hand, miRNAs are known to play an essential role in fine-tuning host immune homeostasis and responses, as miRNA-mediated regulation of gene expression has a profound impact on immune cell development, function, and response to invading pathogens. Interestingly, we recently observed that HHV-6A/6B infection of human thyrocytes and T-lymphocytes profoundly remodulates the expression of cellular miRNAs, inducing specific miRNAs associated to autoimmunity *in vivo* (Caselli et al., 2017), and of transcription factors (unpublished observations).

To study the effects of HHV-6A and -6B on NK cell functions, we analyzed the effect of *in vitro* infection of NK cells on the expression of miRNAs. We also investigated the expression of transcription factors in infected NK cells, in the attempt to further clarify the details of intracellular alterations induced by these viruses with relevance on the immune function.

## Materials and Methods

### Cells and Viruses

The human NK-92 natural killer cell line (ATCC^®^ CRL-2407^TM^) was used for all infection experiments (Gong et al., 1994). Cells were expanded in Alpha Minimum Essential Medium supplemented with 2 mM L-glutamine, 1.5 g/L sodium bicarbonate, 0.1 mM 2-mercaptoethanol, and 20% fetal bovine serum (FBS) (complete medium).

Cell-free virus inocula were obtained as described previously (Caselli et al., 2006; Caruso et al., 2009), and quantified by quantitative real-time PCR (qPCR) (Caselli et al., 2017). All the experiments were performed by using the same virus inoculum, containing 10^10^ genome copies/ml, corresponding to about 10^9^ infecting particles/ml, as previously described (Caselli et al., 2015).

All experiments involving virus production and infection were performed under standard BLS-2 biosafety level.

### NK Cell Infection

NK-92 cells were seeded at optimal density and after 24 h HHV-6A or 6B were added at a 100:1 multiplicity of infection (MOI, virus genomes:cell ratio). Virus adsorption was carried out in a 2% FBS medium for 3 h, then the excess virus was eliminated by centrifugation and washing in PBS, and cells were finally seeded at 0.5 × 10^6^ cells/ml in complete medium with high FBS concentration. Control cells were treated with the same procedure but uninfected. At 1, 2, 3, and 6 days post-infection (d.p.i.) aliquots of cultures were collected and analyzed for virus DNA presence, virus transcription and antigen expression, as well as for expression of miRNAs and transcription factors. Evaluation of cell viability was performed by cell counting after Trypan Blue exclusion test.

### qPCR Analyses of Virus DNA Presence

Total DNA was extracted from the infected or uninfected cells by a commercial kit (Exgene Cell SV kit, GeneAll Biotechnology, Korea), and quantified by spectrophotometric reading at 260 and 280 nm. Virus DNA presence was verified and quantified as previously described by a qPCR detecting the conserved U94 gene of HHV-6A and 6B with equivalent efficiency (Caselli et al., 2017).

### Microarray and RT-qPCR Analyses

RNA was extracted from cells by the miRNeasy kit (Qiagen, Hilden, Germany), allowing the extraction of total RNA including miRNA fraction, as previously described (Caselli et al., 2017). Extracted RNA were devoid of contaminant DNA, as assured by DNase treatment and control β-actin PCR without reverse transcription (Caselli et al., 2012). RNA reverse transcription was performed by the RT2 First strand Kit (Qiagen, Hilden, Germany) for analyses of virus transcripts and human transcription factors, whereas for miRNA analyses the miScript RT kit was used (Qiagen, Hilden, Germany). cDNA aliquots corresponding to 200 ng RNA were used for virus transcription analysis, performed by qPCR detecting the expression of U94, U42 and U22 genes, as previously reported (Caselli et al., 2012). The expression of transcription factors was analyzed by the ‘Human Transcription Factor’ microarray, detecting and quantifying simultaneously 83 different human transcription factors (Qiagen, Hilden, Germany), using 500 ng cDNA as the template.

miRNA analyses were performed on 100 ng of specifically reverse transcribed cDNA, by the ‘Human Inflammatory Response & Autoimmunity’ Microarray (Qiagen, Hilden, Germany), quantifying simultaneously 84 different miRNAs involved in the immune response, and by 20 individual assays chosen to detect and quantify miRNAs specifically involved in the NK cell functions. Namely, the following individual miRNAs were analyzed: miR155^∗^_1, miR155_2, miR146a_1, miR16-1^∗^_1, miR16_2, miR181a^∗^_1, miR181a_2, miR181b_1, miR181b-3p_1, miR10a^∗^_1, miR10a_2, miR150_1, miR150-3p_1, miR27a_1, miR27a^∗^_1, miR27b^∗^_1, miR27b_2, miR223_1, miR223^∗^_1, miR378^∗^_1; miRTC_1, and SNORD61_11 were used as internal controls (all Qiagen, Hilden, Germany).

All qPCR amplification results obtained by transcription factors microarray, miRNA microarray and individual assays, were analyzed and normalized by a specific Qiagen software, to obtain comparable values between control and infected cells at each time post-infection.

### Immunofluorescence Analysis

Immunofluorescence for HHV-6A/6B antigen expression was performed with mouse monoclonal antibodies (mAb) directed against glycoprotein gp116 (late antigen) of HHV-6 A and B (ABI, Columbia, MD, United States), as previously described (Caselli et al., 2006). Briefly, aliquots of infected or uninfected NK-92 cells were collected by centrifugation 10 min at 1000 × *g*, counted, spotted on a glass slide (50,000 cell in 10 μl), dried at room temperature and subsequently stained as described elsewhere (Caselli et al., 2006).

### Statistical Analysis

Statistical analysis of collected data was performed using the Stat View software package (SAS Institute, Inc., Cary, NC, United States). Comparative analysis between individual parameters in infected and control groups was performed by Student’s *t*-test, and *p*-values ≤ 0.01 were considered significant. For multiple comparisons, the Bonferroni correction was applied, and corrected *p*-values (*p_c_*) ≤ 0.01 were considered significant.

## Results

### HHV-6A and 6B Infect Productively NK-92 Cells

**FIGURE 1:**
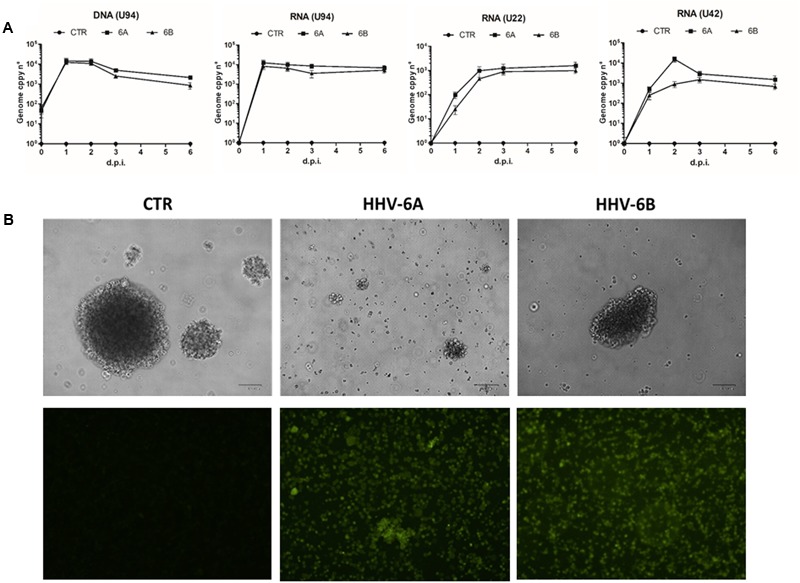
Infection by HHV-6A and 6B in the human NK-92 natural killer cell line. NK-92 cells were infected with HHV-6A or 6B cell-free virus inocula (MOI = 100). **(A)** Virus presence (DNA) and transcription (RNA) were analyzed by qPCR and RT-qPCR for U94, U22, and U42 genes. Results are expressed as genome copy number per 100 ng of DNA or RNA. Results are expressed as mean values ± SD of duplicate samples in three independent experiments. **(B)** *Upper panel*, phase contrast microphotographs of uninfected (CTR) or infected cells at 6 d.p.i., original magnification 40X; *lower panel*, results of IFA assays performed using a mouse mAb recognizing the late gp116 glycoprotein of both virus species. The results shown refer to 6 d.p.i.; original magnification 40X. Size bar = 100 μm.

Both viruses established a productive/lytic infection in NK-92 cells, as shown in **Figure 1**, confirming that human NK cells are permissive to viral infection (Lusso et al., 1993), and showing that the NK-92 cell line could be used for all the subsequent analyses.

In fact, virus DNA was present in infected cells at all time-points post-infection (1 to 6 d.p.i.), as measured by specific U94 qPCR, and the increased genome number, compared to time-point 0, confirmed that virus replication was actually taking place in infected cells. The analysis of virus transcription confirmed the establishment of productive infection in NK cells, as evidenced by the presence of the immediate-early U42 and late U22 transcripts, together with the U94 transcript (which is detected both during lytic and latent HHV-6 infection), at all time-points post-infection (p.i.) (**Figure 1A**). Establishment of lytic infection was confirmed by IFA results, showing abundant expression of the late gp116 envelope glycoprotein at 6 d.p.i. in infected cells (**Figure 1B**).

Infected NK-92 cells appeared damaged and less able to form clusters compared to uninfected controls, with more pronounced effects in the case of HHV-6A, compared to HHV-6B (**Figure 1B**). The virus-induced damages were confirmed by viable cell counts, showing a slight decrease in HHV-6A and 6B infected cells at 1 d.p.i. (-14.5 ± 3.8% compared to control uninfected cells), but a more evident reduction at later time-points, with HHV-6A infection causing a 66.7 ± 9.8% cell loss and HHV-6B a 39.8 ± 6.5% cell loss, at 6 d.p.i., compared to controls.

### miRNA Expression Modulation by HHV-6A and 6B Infection

**FIGURE 2:**
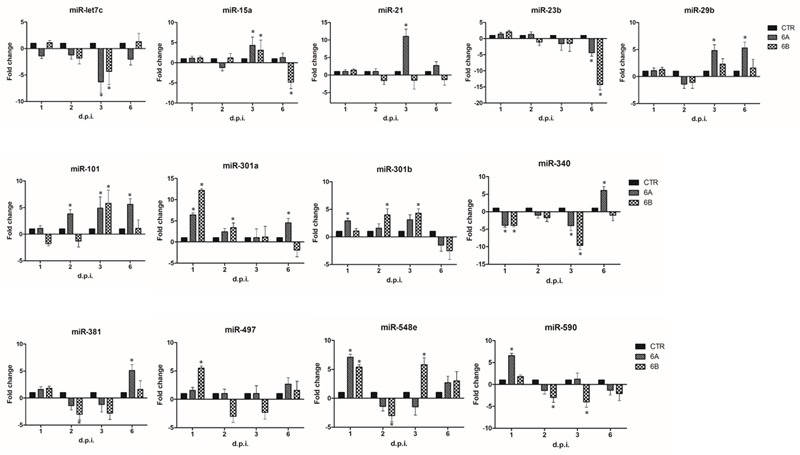
HHV-6A/6B impact on the expression of inflammation and autoimmunity associated miRNAs in NK-92 cells (most affected miRNAs). NK-92 cells were uninfected (control) or infected with HHV-6A or 6B cell-free inocula, then analyzed at 1, 2, 3, and 6 d.p.i. for miRNA expression by a microarray detecting 84 miRNAs. Results represent the fold-changes compared to control values, and are expressed as mean values ± SD (duplicate samples from three independent experiments). Statistically significant alterations, after Bonferroni correction, are marked with asterisks (*p*_c_≤ 0.01).

miRNA expression in infected cells was first studied by a microarray assay simultaneously detecting 84 different miRNAs associated to the development of inflammation and autoimmunity. The results, summarized in Supplementary Table S1, show that both HHV-6A and 6B induce evident alterations in the expression of cellular miRNAs. In particular, 23 miRNAs resulted significantly modulated (p_c_ ≤ 0.01) compared to controls, in at least one time-point p.i. With the aim to highlight only the most prominent effects caused by virus infection, we focused our attention on those miRNAs showing at least fourfold changes compared to controls, arbitrarily chosen as a cut-off value. Thirteen miRNAs were modulated more than fourfold by virus infections, with clear early and late effects and differences between the two viruses (**Figure 2**). In particular, both HHV-6A and 6B induced an early up-regulation of miR-301a and miR-548e (1 d.p.i.), an increase of miR-101 and a decrease of miR-let-7c and miR-340 at 3 d.p.i., and a down-regulation of miR-23 at late time-points p.i. (6 d.p.i.). Other effects were specific for each virus species. Namely, HHV-6A specifically induced an early up-regulation of miR-590 (1 d.p.i.), miR-15a and miR-21 (3 d.p.i.), a sustained up-regulation of miR-29b, miR-101 (3 and 6 d.p.i.), miR-301a and miR-548e (1 and 6 d.p.i.) and a late up-regulation of miR-340 and miR-381 (6 d.p.i.) By contrast, HHV-6B infection specifically up-modulated the expression of miR-301b (2 and 3 d.p.i.) and miR-548e (1 and 3 d.p.i.), whereas it down-regulated miR-590 (2 and 3 d.p.i.) and miR-15a (6 d.p.i.).

Since microarray assays did not include important miRNAs known for their role in NK cell development and function, we analyzed by individual assays the following miRNAs: miR10, miR16, miR27, miR146, miR150, miR155, miR181, miR223, miR378, chosen as they have already been reported to be associated with maturation and activation of NK cells (Liu et al., 2012; Beaulieu et al., 2013; Sullivan et al., 2013).

**FIGURE 3:**
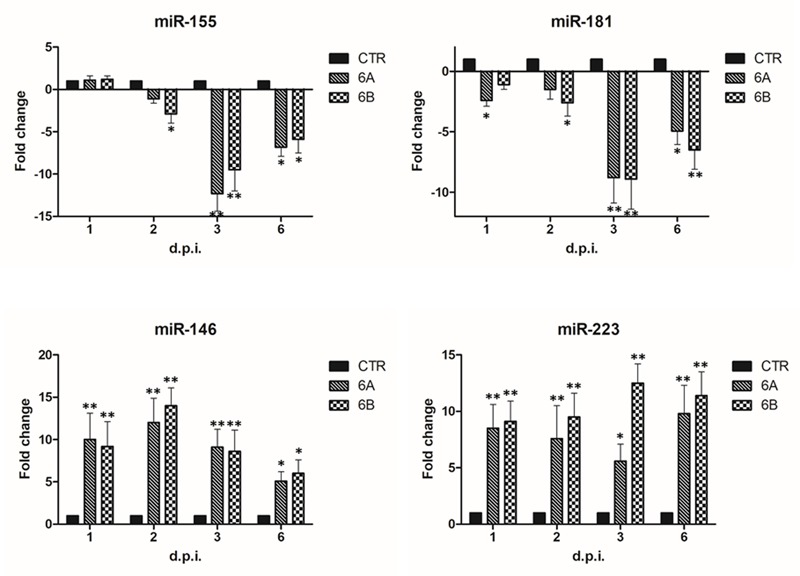
Modulation induced by HHV-6A and 6B infection on specific miRNAs associated to NK cell functions. NK-92 cells were uninfected (control) or infected with HHV-6A or 6B cell-free inocula, then analyzed at 1, 2, 3, and 6 d.p.i. for miRNA expression by individual assays detecting miRNAs already known for their role in NK cell maturation, activation of effector functions. Results are expressed as fold-change ± SD, compared to control values, and represent the mean value of duplicate samples from three independent experiments. Statistically significant values are marked with asterisks (^∗^*p* ≤ 0.01; ^∗∗^*p* < 0.001).

The results showed that four additional miRNAs were altered by HHV-6A and 6B infection (**Figure 3**), as both viruses, although to a different extent, induced a significant decrease (up to 12-fold compared to controls; *p* ≤ 0.001) of miR-155 and miR-181, which are respectively involved in cytotoxicity and maturation of NK cells, and a concomitant significant increase (up to 14-fold; *p* ≤ 0.001) in the expression of miR-146 and miR-223, which are associated to NK cell effector functions, including IFNγ and TNFα production.

### Transcription Factors Modulation by HHV-6A/6B Infection

We analyzed the impact of HHV-6A and -6B infection on the expression of human transcription factors, with the simultaneous analysis of 83 different transcription factors by microarray.

**FIGURE 4:**
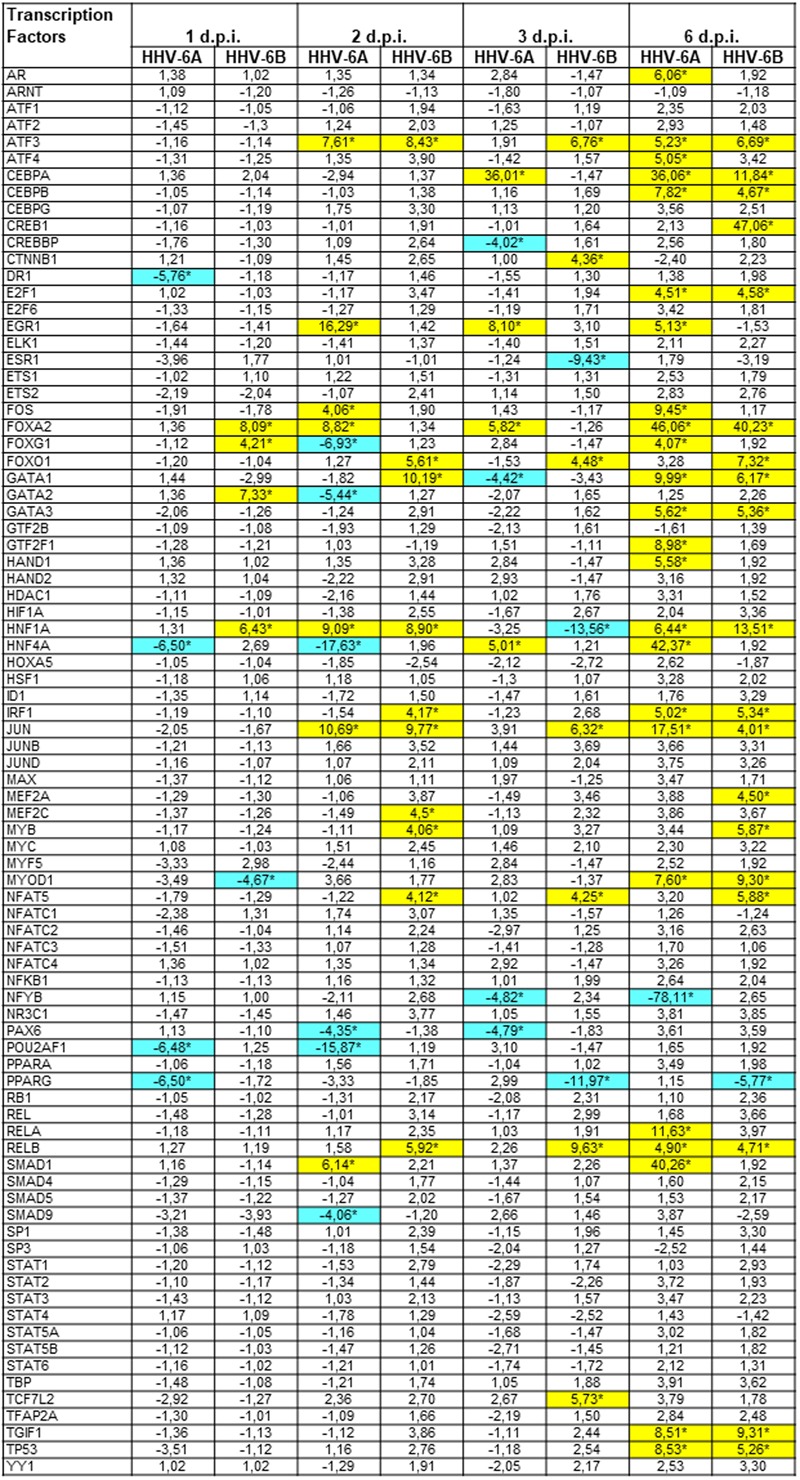
Modulation induced by HHV-6A and 6B infection on human transcription factors mRNAs in NK-92 cells. NK-92 cells were uninfected (control) or infected with HHV-6A or 6B cell-free inocula, then analyzed at 1, 2, 3, and 6 d.p.i. for mRNA expression by a microarray detecting 83 transcription factors. Results are expressed as fold-change compared to control values, and represent the mean value of duplicate samples from three independent experiments. Statistically significant differences after Bonferroni correction are marked with asterisks (*p*_c_≤ 0.01) and highlighted by colors (yellow ≥ 4-fold up-regulations; blue ≥ 4-fold down-regulations). Exact *p*_c_ values of all significant parameters varied from 0.005 to 0.01.

The results showed that HHV-6 infection strongly modulates the expression of transcription factors (**Figure 4**). In fact, more than 30 transcription factors were significantly up or down-modulated compared to controls, at different time-points p.i (p_c_ < 0.01). In particular, the remodulation of transcription factors was evident at late time-points p.i., whereas at early time-points p.i. viral infection had lower impact, with clear differences between the two species. In fact, while HHV-6A was essentially down-modulating a few transcription factors at 1 d.p.i. (DR1, HNF4A, POU2AF1, PPARγ), HHV-6B had an essentially up-regulating effect, increasing the expression of FOXA2, FOXG1, GATA2, HNF1A, and decreasing only the expression of MYOD1. By contrast, at 6 d.p.i. both HHV-6A and HHV-6B infections resulted in the up-modulation of several transcription factors, perhaps related to the evident CPE and cell lysis induced by viruses in the infected NK cells.

Both viruses induced the up-regulation of ATF3, CEBPA, CEBPB, JUN and FOXA2 factors, and the down-modulation of PPARγ factor. However, other factors were modulated by only one virus. For example, EGR1 and FOS were up-regulated only by HHV-6A (at 2, 3, 6 d.p.i.), which also specifically induced the decrease of POU2AF1 expression (at 1, 2 d.p.i.) and NFYB (at 2, 3, 6 d.p.i.). Instead, HHV-6B induced an increase in FOXO1 (2, 3 and 6 d.p.i.) and CREB1 (6 d.p.i.) expression and a down-regulation of ESR1 at 3 d.p.i. Other transcription factors displayed a biphasic modulation, as they were differently regulated by viruses at early and late time-points post-infection: the GATA family, HNF1A and HNF4A.

## Discussion

In their continuous interaction with the host immune system, herpesviruses have developed a large array of strategies to escape the host defense mechanisms. In particular, although different for many biological and pathological aspects, both HHV-6A and 6B display a strong tropism toward lymphocytes, causing important modifications and cytopathic effects in infected cells. Both virus species infect preferentially CD4+ T cells, and HHV-6A productively infects also different types of cytotoxic effectors, including CD8+ T cells, γδT cells, and NK cells (Lusso et al., 1993; Caselli and Di Luca, 2007). By contrast, HHV-6B tropism seems to be more restricted compared to HHV-6A, infecting poorly or at all cytotoxic effector cells (Lusso et al., 1991), but it was recently shown to down-modulate the activating NK cell ligands in SupT1 T cells, impairing the recognition of virus-infected cells by NK cells (Schmiedel et al., 2016).

Although the importance of the interplay between immune cells and HHV-6A/6B has been recognized as a key factor in viral immune evasion strategies and in the development of associated pathologies, few mechanisms have been elucidated. Our study shows for the first time that HHV-6A and HHV-6B productively infect NK cells and induce evident modifications in the expression of two sets of intracellular mediators of effector functions: miRNAs and transcription factors. In particular, microarray data showed that HHV-6A and 6B manipulate the expression of cellular miRNAs in NK cells, inducing both common and species-specific effects.

The results show that the two viruses may induce a substantial alteration in NK cell functions, as most of the modulated miRNAs are involved in important immune functions. In fact, both HHV-6A and 6B down-modulate miR-let-7, which is highly expressed in NK cells, CD4+ and CD8+ T cells. Interestingly, this miRNA was reported to be down-modulated also by murine Cytomegalovirus (Beaulieu et al., 2013) and HIV-1 infection (Swaminathan et al., 2012), where it was associated with an increase of anti-inflammatory IL-10, thus providing viruses with an important immune escape mechanism. Similarly, decrease in miR-340 expression might protect viruses from the immune response, as miR-340 directly targets the Th2 effector proinflammatory cytokine IL-4 (Podshivalova and Salomon, 2013; Kim et al., 2016). Studies have in fact reported that IL-4 can enhance viral virulence in diverse models, including HSV-1 eye disease, possibly by suppressing cytotoxic lymphocyte response (Ghiasi et al., 1999; Jackson et al., 2001; Kerr et al., 2004). On the contrary, the increase of miR-301a (up-regulated by both viruses) has been reported to block the IRF1 innate immune response against Japanese encephalitis virus and might therefore favor HHV-6 pathogenesis by inhibiting IFNβ production (Hazra et al., 2017). Interestingly, miR-301 was also up-regulated in T cells in the central nervous system of animals with experimental autoimmune encephalomyelitis (Mycko et al., 2012), an animal model for the process of autoimmune demyelination occurring during multiple sclerosis, a disease with a possible association with HHV-6 infection (Leibovitch and Jacobson, 2014). The up-regulated miR-101 and miR-381 are involved in the polarization and activation of the cells of the innate immune compartment, regulating the inflammatory response (Zhu et al., 2010; Essandoh et al., 2016; Wen et al., 2016); the increased miR-548 may represent a mechanism facilitating viral pathogenesis as it negatively correlates with IFNγR1 levels (Xing et al., 2014). miR-21 and miR-590, up-regulated by HHV-6A, are involved in differentiation and activation of T cells, particularly during autoimmunity (Sousa et al., 2017), with miR-590 also down-regulating critical genes of signaling pathways similar in cancer and inflammation (Sheikholeslami et al., 2017). miR-29 and miR-15, both increased by HHV-6A infection, have a specific role in regulating the cytotoxic activity of NK cells, inhibiting the production of IFNγ by directly targeting the 3′ UTR of its mRNA (Ma et al., 2011; Leong et al., 2014).

Interestingly, HHV-6B, but not HHV-6A, infection down-modulated miR-590 and miR-15, suggesting that the two species might impact differently on NK cell functions. Notably, however, both species significantly decreased miR-155, important in the effector functions of NK cells as it stimulates IFNγ production in activated NK cells (Trotta et al., 2012), and miR-181, essential for the correct maturation of NK cells (Cichocki et al., 2011). Both viruses induced an increase of miR-146, which negatively regulates NK activity by inhibiting cytotoxicity, IFNγ and TNFα production (Xu et al., 2016), and of miR-223, which inhibits the production of granzyme B by murine NK cells (Fehniger et al., 2010; Leong et al., 2014). Intriguingly, miR-223 has been associated to the pathogenesis of autoimmune thyroiditis, another disease which has been correlated to HHV-6 infection (Caselli et al., 2012). Interestingly, the modifications of miRNA expression induced by HHV-6A and 6B infections in NK cells were not superimposable to those observed in T cells (Caselli et al., 2017), demonstrating that, as well as species-specific, the effects induced by the two viruses are also cell type-specific, as they impact differently on the expression of miRNAs in different immune cell types.

Our analyses showed also that HHV-6A and 6B modulate the expression of several transcription factors in infected NK cells, with effects shared by both viruses, or specifically induced by only one of them, underlining again the different impact of the two viruses on NK cells. In fact, both viruses induced the up-regulation of ATF3, CEBPA, JUN and FOXA2, and down-regulation of PPARγ factor. Whereas HHV-6A induced an increase in EGR1 and FOS expression and a decrease of POU2AF1 expression, HHV-6B induced an increase in FOXO1 and a down-regulation of ESR1 expression. Although many of the modulated factors are not yet associated to specific functions in NK cells biology, some of them have already been described to have a role in this context, or in closely related immune aspects.

Interestingly, ATF3, upregulated by both viruses, was reported to regulate negatively NK cell functions in MCMV infected mice, by modulating IFNγ expression (Rosenberger et al., 2008). On the other hand, although not yet studied in NK cells, several evidences currently associate PPARγ to Th lymphocyte differentiation, B lymphocyte effector functions and cytokine expression (da Rocha Junior et al., 2013). POU2AF1, down-modulated by HHV-6A, was recently reported to induce upregulation of host defense genes, including IL-6, in airway epithelium (Zhou et al., 2016). FOXO1 and ESR1, respectively increased and decreased by HHV-6B, are important regulators of the immune response, being FOXO1 a negative regulator of NK cell maturation and functions (Deng et al., 2015), whereas ESR1 has been associated to regulation of inflammatory pathways of innate immune cells (Kovats, 2015). Other factors, including JUN, FOS, CEBPA, are involved in several biological processes as regulators of cell cycle progression, hematopoietic cell differentiation and apoptosis (Foletta et al., 1998; Wisdom et al., 1999; Ponti et al., 2002; Healy et al., 2013), and might be studied in detail in the NK cell context.

Overall, our data show for the first time that infection by HHV-6A and 6B profoundly impacts the intracellular environment of infected NK cells, likely inducing biological effects helping the viruses to escape the innate immune response. Although the two different HHV-6 species induce many common effects, our data also show species-specific effects on miRNAs and transcription factors expression. The differences might possibly result in a different biological impact of the two viruses, potentially associated to specific pathological conditions.

## Conclusion

HHV-6A and 6B induce significant alterations on the expression of several miRNAs and transcription factors in infected NK cells. These alterations might lead to important modifications in NK cell ability to control HHV-6 infections, enabling immune evasion and facilitating viral replication cycle, and impacting on NK cell involvement in inflammation and autoimmune reactions.

Functional studies should be conducted to investigate the role of the factors altered by HHV-6 infection, and to evaluate their possible implication in pathological alterations and disease progression.

## Author Contributions

RR contributed to the conception of the work and data analysis. IS, MD, and DB contributed to data collection and analysis. DDL contributed to data interpretation and critical revision of the article. EC contributed to the conception of the work, data acquisition and analysis, and writing the article.

## Conflict of Interest Statement

The authors declare that the research was conducted in the absence of any commercial or financial relationships that could be construed as a potential conflict of interest.

## References

[B1] Ablashi D. Agut H. Alvarez-Lafuente R. Clark D. A. Dewhurst S. DiLuca D. (2014). Classification of HHV-6A and HHV-6B as distinct viruses. *Arch. Virol.* 159 863–870. 10.1007/s00705-013-1902-5 24193951 PMC4750402

[B2] Arens R. (2012). Rational design of vaccines: learning from immune evasion mechanisms of persistent viruses and tumors. *Adv. Immunol.* 114 217–243. 10.1016/B978-0-12-396548-6.00009-3 22449784

[B3] Beaulieu A. M. Bezman N. A. Lee J. E. Matloubian M. Sun J. C. Lanier L. L. (2013). MicroRNA function in NK-cell biology. *Immunol. Rev.* 253 40–52. 10.1111/imr.12045 23550637 PMC3621029

[B4] Biron C. A. Byron K. S. Sullivan J. L. (1989). Severe herpesvirus infections in an adolescent without natural killer cells. *N. Engl. J. Med.* 320 1731–1735. 10.1056/NEJM198906293202605 2543925

[B5] Caruso A. Caselli E. Fiorentini S. Rotola A. Prandini A. Garrafa E. (2009). U94 of human herpesvirus 6 inhibits in vitro angiogenesis and lymphangiogenesis. *Proc. Natl. Acad. Sci. U.S.A.* 106 20446–20451. 10.1073/pnas.0905535106 19918067 PMC2787175

[B6] Caselli E. Bracci A. Galvan M. Boni M. Rotola A. Bergamini C. (2006). Human herpesvirus 6 (HHV-6) U94/REP protein inhibits betaherpesvirus replication. *Virology* 346 402–414. 10.1016/j.virol.2005.11.018 16368124

[B7] Caselli E. Campioni D. Cavazzini F. Gentili V. Bortolotti D. Cuneo A. (2015). Acute human herpesvirus-6A infection of human mesothelial cells modulates HLA molecules. *Arch. Virol.* 160 2141–2149. 10.1007/s00705-015-2490-3 26085284

[B8] Caselli E. D’Accolti M. Soffritti I. Zatelli M. C. Rossi R. Degli Uberti E. (2017). HHV-6A in vitro infection of thyrocytes and T cells alters the expression of miRNA associated to autoimmune thyroiditis. *Virol. J.* 14:3. 10.1186/s12985-016-0672-6 28081700 PMC5234148

[B9] Caselli E. Di Luca D. (2007). Molecular biology and clinical associations of Roseoloviruses human herpesvirus 6 and human herpesvirus 7. *New Microbiol.* 30 173–187. 17802896

[B10] Caselli E. Zatelli M. C. Rizzo R. Benedetti S. Martorelli D. Trasforini G. (2012). Virologic and immunologic evidence supporting an association between HHV-6 and hashimoto’s thyroiditis. *PLOS Pathog.* 8:e1002951. 10.1371/journal.ppat.1002951 23055929 PMC3464215

[B11] Cichocki F. Felices M. McCullar V. Presnell S. R. Al-Attar A. Lutz C. T. (2011). Cutting edge: microRNA-181 promotes human NK cell development by regulating Notch signaling. *J. Immunol.* 187 6171–6175. 10.4049/jimmunol.1100835 22084432 PMC3237765

[B12] Cooper M. A. Fehniger T. A. Caligiuri M. A. (2001). The biology of human natural killer-cell subsets. *Trends Immunol.* 22 633–640. 10.1016/S1471-4906(01)02060-9 11698225

[B13] da Rocha Junior L. F. Dantas A. T. Duarte A. L. de Melo Rego M. J. Pitta Ida R. Pitta M. G. (2013). PPARgamma agonists in adaptive immunity: what do immune disorders and their models have to tell us? *PPAR Res.* 2013:519724. 10.1155/2013/519724 23983678 PMC3747405

[B14] Dagna L. Pritchett J. C. Lusso P. (2013). Immunomodulation and immunosuppression by human herpesvirus 6A and 6B. *Future Virol.* 8 273–287. 10.2217/fvl.13.7 24163703 PMC3806647

[B15] Deng Y. Kerdiles Y. Chu J. Yuan S. Wang Y. Chen X. (2015). Transcription factor Foxo1 is a negative regulator of natural killer cell maturation and function. *Immunity* 42 457–470. 10.1016/j.immuni.2015.02.006 25769609 PMC4400836

[B16] Essandoh K. Li Y. Huo J. Fan G. C. (2016). MiRNA-mediated macrophage polarization and its potential role in the regulation of inflammatory response. *Shock* 46 122–131. 10.1097/SHK.0000000000000604 26954942 PMC4949115

[B17] Fehniger T. A. Wylie T. Germino E. Leong J. W. Magrini V. J. Koul S. (2010). Next-generation sequencing identifies the natural killer cell microRNA transcriptome. *Genome Res.* 20 1590–1604. 10.1101/gr.107995.110 20935160 PMC2963822

[B18] Fleisher G. Starr S. Koven N. Kamiya H. Douglas S. D. Henle W. (1982). A non-x-linked syndrome with susceptibility to severe Epstein-Barr virus infections. *J. Pediatr.* 100 727–730. 10.1016/S0022-3476(82)80572-6 6279813

[B19] Foletta V. C. Segal D. H. Cohen D. R. (1998). Transcriptional regulation in the immune system: all roads lead to AP-1. *J. Leukoc. Biol.* 63 139–152. 9468273 10.1002/jlb.63.2.139

[B20] Ghiasi H. Cai S. Slanina S. M. Perng G. C. Nesburn A. B. Wechsler S. L. (1999). The role of interleukin (IL)-2 and IL-4 in herpes simplex virus type 1 ocular replication and eye disease. *J. Infect. Dis.* 179 1086–1093. 10.1086/314736 10191208

[B21] Gong J. H. Maki G. Klingemann H. G. (1994). Characterization of a human cell line (NK-92) with phenotypical and functional characteristics of activated natural killer cells. *Leukemia* 8 652–658. 8152260

[B22] Hazra B. Kumawat K. L. Basu A. (2017). The host microRNA miR-301a blocks the IRF1-mediated neuronal innate immune response to Japanese encephalitis virus infection. *Sci. Signal.* 10:eaaf5185. 10.1126/scisignal.aaf5185 28196914

[B23] Healy S. Khan P. Davie J. R. (2013). Immediate early response genes and cell transformation. *Pharmacol. Ther.* 137 64–77. 10.1016/j.pharmthera.2012.09.001 22983151

[B24] Hirata Y. Kondo K. Yamanishi K. (2001). Human herpesvirus 6 downregulates major histocompatibility complex class I in dendritic cells. *J. Med. Virol.* 65 576–583. 10.1002/jmv.2075 11596096

[B25] Jackson R. J. Ramsay A. J. Christensen C. D. Beaton S. Hall D. F. Ramshaw I. A. (2001). Expression of mouse interleukin-4 by a recombinant ectromelia virus suppresses cytolytic lymphocyte responses and overcomes genetic resistance to mousepox. *J. Virol.* 75 1205–1210. 10.1128/JVI.75.3.1205-1210.2001 11152493 PMC114026

[B26] Kerr P. J. Perkins H. D. Inglis B. Stagg R. McLaughlin E. Collins S. V. (2004). Expression of rabbit IL-4 by recombinant myxoma viruses enhances virulence and overcomes genetic resistance to myxomatosis. *Virology* 324 117–128. 10.1016/j.virol.2004.02.031 15183059

[B27] Kim E. S. Choi Y. E. Hwang S. J. Han Y. H. Park M. J. Bae I. H. (2016). IL-4, a direct target of miR-340/429, is involved in radiation-induced aggressive tumor behavior in human carcinoma cells. *Oncotarget* 7 86836–86856. 10.18632/oncotarget.13561 27895317 PMC5349958

[B28] Kovats S. (2015). Estrogen receptors regulate innate immune cells and signaling pathways. *Cell Immunol.* 294 63–69. 10.1016/j.cellimm.2015.01.018 25682174 PMC4380804

[B29] Leibovitch E. C. Jacobson S. (2014). Evidence linking HHV-6 with multiple sclerosis: an update. *Curr. Opin. Virol.* 9 127–133. 10.1016/j.coviro.2014.09.016 25462444 PMC4269240

[B30] Leong J. W. Sullivan R. P. Fehniger T. A. (2014). microRNA management of NK-cell developmental and functional programs. *Eur. J. Immunol.* 44 2862–2868. 10.1002/eji.201444798 25142111 PMC4228684

[B31] Liu X. Wang Y. Sun Q. Yan J. Huang J. Zhu S. (2012). Identification of microRNA transcriptome involved in human natural killer cell activation. *Immunol. Lett.* 143 208–217. 10.1016/j.imlet.2012.02.014 22701882

[B32] Lusso P. (2006). HHV-6 and the immune system: mechanisms of immunomodulation and viral escape. *J. Clin. Virol.* 37(Suppl. 1) S4–S10. 10.1016/S1386-6532(06)70004-X 17276368

[B33] Lusso P. De Maria A. Malnati M. Lori F. DeRocco S. E. Baseler M. (1991). Induction of CD4 and susceptibility to HIV-1 infection in human CD8+ T lymphocytes by human herpesvirus 6. *Nature* 349 533–535. 10.1038/349533a0 1846951

[B34] Lusso P. Malnati M. S. Garzino-Demo A. Crowley R. W. Long E. O. Gallo R. C. (1993). Infection of natural killer cells by human herpesvirus 6. *Nature* 362 458–462. 10.1038/362458a0 7681936

[B35] Ma F. Xu S. Liu X. Zhang Q. Xu X. Liu M. (2011). The microRNA miR-29 controls innate and adaptive immune responses to intracellular bacterial infection by targeting interferon-gamma. *Nat. Immunol.* 12 861–869. 10.1038/ni.2073 21785411

[B36] Mycko M. P. Cichalewska M. Machlanska A. Cwiklinska H. Mariasiewicz M. Selmaj K. W. (2012). MicroRNA-301a regulation of a T-helper 17 immune response controls autoimmune demyelination. *Proc. Natl. Acad. Sci. U.S.A.* 109 E1248–E1257. 10.1073/pnas.1114325109 22517757 PMC3356660

[B37] Orange J. S. (2002). Human natural killer cell deficiencies and susceptibility to infection. *Microbes Infect.* 4 1545–1558. 10.1016/S1286-4579(02)00038-2 12505527

[B38] Podshivalova K. Salomon D. R. (2013). MicroRNA regulation of T-lymphocyte immunity: modulation of molecular networks responsible for T-cell activation, differentiation, and development. *Crit. Rev. Immunol.* 33 435–476. 10.1615/CritRevImmunol.2013006858 24099302 PMC4185288

[B39] Poggi A. Zocchi M. R. (2014). NK cell autoreactivity and autoimmune diseases. *Front. Immunol.* 5:27. 10.3389/fimmu.2014.00027 24550913 PMC3912987

[B40] Ponti C. Gibellini D. Boin F. Melloni E. Manzoli F. A. Cocco L. (2002). Role of CREB transcription factor in c-fos activation in natural killer cells. *Eur. J. Immunol.* 32 3358–3365. 10.1002/1521-4141(200212)32:12<3358::AID-IMMU3358>3.0.CO;2-Q 12432566

[B41] Rosenberger C. M. Clark A. E. Treuting P. M. Johnson C. D. Aderem A. (2008). ATF3 regulates MCMV infection in mice by modulating IFN-gamma expression in natural killer cells. *Proc. Natl. Acad. Sci. U.S.A.* 105 2544–2549. 10.1073/pnas.0712182105 18268321 PMC2268173

[B42] Schleinitz N. Vely F. Harle J. R. Vivier E. (2010). Natural killer cells in human autoimmune diseases. *Immunology* 131 451–458. 10.1111/j.1365-2567.2010.03360.x 21039469 PMC2999796

[B43] Schmiedel D. Tai J. Levi-Schaffer F. Dovrat S. Mandelboim O. (2016). Human herpesvirus 6B downregulates expression of activating ligands during lytic infection to escape elimination by natural killer cells. *J. Virol.* 90 9608–9617. 10.1128/JVI.01164-16 27535049 PMC5068514

[B44] Sheikholeslami A. Nabiuni M. Arefian E. (2017). Suppressing the molecular signaling pathways involved in inflammation and cancer in breast cancer cell lines MDA-MB-231 and MCF-7 by miR-590. *Tumour. Biol.* 39:1010428317697570. 10.1177/1010428317697570 28443471

[B45] Smith A. Santoro F. Di Lullo G. Dagna L. Verani A. Lusso P. (2003). Selective suppression of IL-12 production by human herpesvirus 6. *Blood* 102 2877–2884. 10.1182/blood-2002-10-3152 12829600

[B46] Sousa I. G. do Almo M. M. Simi K. C. Bezerra M. A. Andrade R. V. Maranhao A. Q. (2017). MicroRNA expression profiles in human CD3+ T cells following stimulation with anti-human CD3 antibodies. *BMC Res. Notes* 10:124. 10.1186/s13104-017-2442-y 28292330 PMC5351193

[B47] Sullivan R. P. Leong J. W. Fehniger T. A. (2013). MicroRNA regulation of natural killer cells. *Front. Immunol.* 4:44. 10.3389/fimmu.2013.00044 23450173 PMC3584293

[B48] Swaminathan S. Suzuki K. Seddiki N. Kaplan W. Cowley M. J. Hood C. L. (2012). Differential regulation of the Let-7 family of microRNAs in CD4+ T cells alters IL-10 expression. *J. Immunol.* 188 6238–6246. 10.4049/jimmunol.1101196 22586040

[B49] Trotta R. Chen L. Ciarlariello D. Josyula S. Mao C. Costinean S. (2012). miR-155 regulates IFN-gamma production in natural killer cells. *Blood* 119 3478–3485. 10.1182/blood-2011-12-398099 22378844 PMC3325038

[B50] Vivier E. Tomasello E. Baratin M. Walzer T. Ugolini S. (2008). Functions of natural killer cells. *Nat. Immunol.* 9 503–510. 10.1038/ni1582 18425107

[B51] Vossen M. T. Westerhout E. M. Soderberg-Naucler C. Wiertz E. J. (2002). Viral immune evasion: a masterpiece of evolution. *Immunogenetics* 54 527–542. 10.1007/s00251-002-0493-1 12439615

[B52] Wen Q. Zhou C. Xiong W. Su J. He J. Zhang S. (2016). MiR-381-3p regulates the antigen-presenting capability of dendritic cells and represses antituberculosis cellular immune responses by targeting CD1c. *J. Immunol.* 197 580–589. 10.4049/jimmunol.1500481 27296666

[B53] Wisdom R. Johnson R. S. Moore C. (1999). c-Jun regulates cell cycle progression and apoptosis by distinct mechanisms. *EMBO J.* 18 188–197. 10.1093/emboj/18.1.188 9878062 PMC1171114

[B54] Xing T. J. Xu H. T. Yu W. Q. Wang B. Zhang J. (2014). MiRNA-548ah, a potential molecule associated with transition from immune tolerance to immune activation of chronic hepatitis B. *Int. J. Mol. Sci.* 15 14411–14426. 10.3390/ijms150814411 25196343 PMC4159859

[B55] Xu D. Han Q. Hou Z. Zhang C. Zhang J. (2016). miR-146a negatively regulates NK cell functions via STAT1 signaling. *Cell Mol. Immunol.* 14 712–720. 10.1038/cmi.2015.113 26996068 PMC5549603

[B56] Yamanishi K. Okuno T. Shiraki K. Takahashi M. Kondo T. Asano Y. (1988). Identification of human herpesvirus-6 as a causal agent for exanthem subitum. *Lancet* 1 1065–1067. 10.1016/S0140-6736(88)91893-4 2896909

[B57] Zhou H. Brekman A. Zuo W. L. Ou X. Shaykhiev R. Agosto-Perez F. J. (2016). POU2AF1 functions in the human airway epithelium to regulate expression of host defense genes. *J. Immunol.* 196 3159–3167. 10.4049/jimmunol.1502400 26927796 PMC4799774

[B58] Zhu Q. Y. Liu Q. Chen J. X. Lan K. Ge B. X. (2010). MicroRNA-101 targets MAPK phosphatase-1 to regulate the activation of MAPKs in macrophages. *J. Immunol.* 185 7435–7442. 10.4049/jimmunol.1000798 21068409

