# Supplementary material for: HHV-6A/6B Infection of NK Cells Modulates the Expression of miRNAs and Transcription Factors Potentially Associated to Impaired NK Activity
Source: Front Microbiol. 2017 Oct 31;8:2143. doi: 10.3389/fmicb.2017.02143 (PMC5671584; doi:10.3389/fmicb.2017.02143)
Supplement: Supplementary file 1 [file Table_1.PDF]

Table S1. HHV-6A/6B impact on the expression of inflammatory/autoimmunity miRNAs in NK cells.

	1 d.p.i.		2 d.p.i.		3 d.p.i.		6 d.p.i.	
**miR**	**HHV-6A**	**HHV-6B**	**HHV-6A**	**HHV-6B**	**HHV-6A**	**HHV-6B**	**HHV-6A**	**HHV-6B**
miR-let-7a-5p	1,45	1,91	-1,09	-1,85	-1,61	-1,95	-1,61	-2,32
miR-let-7b-5p	1,56	1,83	-1,44	-2,99	1,01	1,34	2,65	1,72
miR-let-7c-5p	-1,41	1,14	-1,21	-1,78	-6,28* (0.002)	-4,34* (0.005)	-2,01	1,33
miR-let-7d-5p	1,89	2,42	-1,08	-1,85	-3,34* (0.01)	-1,90	-1,12	-2,65
miR-let-7e-5p	1,21	1,78	1,31	-1,48	-2,13	-1,65	-1,21	-1,89
miR-let-7f-5p	1,13	1,61	-1,17	-2,19	-1,87	-1,36	-1,00	-1,97
miR-let-7g-5p	1,14	1,32	1,03	-1,72	-1,93	-1,49	-1,36	-1,96
miR-let-7i-5p	1,24	1,60	1,26	-1,02	1,46	1,55	-1,04	-1,22
miR-9-5p	1,62	1,13	-1,38	-2,81	1,31	-1,11	2,38	1,16
miR-15a-5p	1,09	1,21	-1,22	1,17	4,28* (0.005)	3,19* (0.01)	1,32	-4,77* (0.005)
miR-15b-5p	1,11	1,54	1,09	-2,02	-1,05	-1,62	-1,71	-3,02
miR-16-5p	1,13	1,21	1,11	-1,20	-1,35	-1,15	-1,12	-2,37
miR-17-5p	-1,10	1,01	1,41	1,21	1,03	1,16	1,48	-2,18
miR-19a-3p	-1,17	-1,32	2,62	2,69	1,59	1,87	1,35	-2,69
miR-19b-3p	-1,41	-2,57	2,58	2,19	1,54	2,06	1,06	-3,26
miR-20a-5p	1,17	1,26	1,45	1,13	1,24	1,28	1,43	-1,99
miR-20b-5p	1,05	1,05	1,33	1,09	-1,14	1,30	1,55	-1,97
miR-21-5p	1,03	1,41	1,01	-1,55	11,01* (0.001)	-1,49	2,65	-1,26
miR-23a-3p	1,29	1,81	-1,01	-1,95	-1,48	-1,73	-1,1755	-2,04
miR-23b-3p	1,43	2,15	1,29	-1,07	-1,62	-1,53	-4,42* (0.05)	-14,27* (0.001)
miR-29a-3p	1,03	-1,01	1,36	1,18	-1,53	-1,73	1,3348	-2,62
miR-29b-3p	1,08	1,27	-1,43	-1,09	4,82* (0.01)	2,27	5,28* (0.08)	1,57
miR-29c-3p	1,13	1,14	1,25	-1,01	-1,03	-1,22	1,50	-2,34
miR-30a-5p	1,36	1,55	1,77	-1,22	1,25	-1,50	1,19	-3,54* (0.009)
miR-30b-5p	-1,19	1,09	1,38	-1,16	-1,15	-1,91	-2,02	-2,61
miR-30c-5p	1,02	1,34	1,34	-1,41	-1,71	-1,87	1,11	-2,34
miR-30d-5p	1,39	1,51	2,25	1,07	1,33	1,29	1,27	-3,35
miR-30e-5p	-1,41	-1,32	-1,27	-1,26	1,01	-1,55	1,70	-1,98
miR-34a-5p	1,26	1,83	-1,41	1,79	2,22	-2,86	1,01	-1,67
miR-34c-5p	1,51	1,78	-1,14	-2,54	1,01	-2,35	2,59	1,56
miR-39-3p	1,57	1,83	-1,44	-2,98	1,00	-2,29	2,65	1,56
miR-39-3p	1,51	1,18	-1,54	-2,51	1,01	-2,21	2,24	1,46
miR-93-5p	1,10	1,22	-1,03	-1,23	-1,17	-1,32	1,51	-3,54* (0.01)
miR-98-5p	1,10	1,95	1,18	-1,99	-1,21	-1,09	1,54	-1,59
miR-101-3p	1,11	-1,78	3,75* (0.01)	-1,28	4,92* (0.008)	5,75* (0.005)	5,64* (0.007)	1,07
miR-106b-5p	3,09	1,07	1,38	1,05	1,03	-1,13	1,37	-2,35
miR-125a-5p	1,56	1,83	-1,44	-2,98	1,01	-2,29	2,65	1,56
miR-125b-5p	1,57	1,81	-1,45	-2,99* (0.01)	1,02	-2,32	2,62	1,37
miR-128-3p	1,47	1,82	1,09	-1,74	1,34	-1,53	-1,39	-3,15
miR-130a-3p	1,58	1,78	-1,56	-3,24* (0.009)	1,01	-1,25	2,58	1,53
miR-130b-3p	-1,8	-1,20	1,82	1,16	1,00	-1,19	2,14	-1,55
miR-1324	1,51	1,56	-1,44	-2,99* (0.009)	1,01	-2,29	2,28	1,26
miR-144-3p	1,53	1,42	-1,25	-2,85	1,05	-2,91	2,54	1,63

miR-145-5p	1,32	1,72	-1,58	-2,89	1,07	-2,37	2,47	1,39
miR-181a-5p	1,42	1,25	-1,22	-1,47	1,12	-1,04	-1,07	-1,26
miR-181b-5p	1,21	1,51	-1,47	-2,47	-1,49	-1,63	1,06	-2,06
miR-181c-5p	-1,14	1,49	1,07	-1,06	1,04	-1,31	-1,09	-3,04
miR-181d-5p	-2,42	-2,14	-1,08	-1,07	-3,77* (0.003)	-3,94* (0.003)	-3,13	-3,90* (0.002)
miR-186-5p	-1,41	-1,01	-1,46	-2,52	-1,13	-1,20	-1,48	-3,22
miR-195-5p	1,22	1,31	1,17	-1,21	1,02	-1,01	1,32	-1,83
miR-202-3p	1,57	1,83	-1,44	-2,98	1,01	-2,29	2,65	2,02
miR-211-5p	1,57	1,83	-1,44	-2,98	1,01	-2,39	2,59	1,56
miR-300	1,57	1,83	-1,44	-2,98	1,01	-2,92	2,68	1,55
miR-301a-3p	6,39* (0.002)	12,26* (0.001)	2,39	3,37* (0.01)	1,04	1,24	4,49* (0.005)	-1,92
miR-301b-3p	2,90* (0.009)	1,09	1,64	4,02* (0.005)	3,25	4,27* (0.005)	-1,54	-2,45
miR-302a-3p	1,57	1,56	1,08	-2,98* (0.01)	1,01	-2,21	2,61	1,25
miR-302b-3p	1,56	1,45	-1,24	-2,58	1,05	-2,29	2,73	1,52
miR-302c-3p	1,52	1,78	-1,45	-2,75	1,03	-2,92	2,39	1,61
miR-340-5p	-3,91* (0.006)	-3,94* (0.005)	-1,04	-1,73	-4,14* (0.005)	-9,62* (0.001)	6,09* (0.003)	-1,02
miR-372-3p	1,53	1,25	-1,32	-2,37	1,08	-2,41	2,64	2,14
miR-373-3p	1,56	1,57	-1,24	-2,99	1,14	-2,28	2,61	1,21
miR-374a-5p	-1,03	1,12	1,45	1,02	-1,23	-1,44	1,24	-1,83
miR-381-3p	1,58	1,79	-1,44	-2,98* (0.01)	-1,21	-2,80	5,08* (0.002)	1,63
miR-410-3p	1,43	1,52	-1,38	-2,79	1,01	-2,29	2,78	1,09
miR-424-5p	-1,13	1,04	1,47	1,16	-1,19	-2,70	1,87	-2,18
miR-449a	-1,75	1,32	2,69	1,21	1,04	-2,31	2,25	1,18
miR-449b-5p	1,57	1,83	-1,44	-1,92	1,09	-2,43	2,49	1,00
miR-454-3p	-1,06	1,12	-1,28	-2,36	-1,60	-2,08	1,03	-3,28* (0.01)
miR-497-5p	1,57	5,48* (0.01)	1,04	-2,98	1,09	-2,35	2,68	1,60
miR-511-5p	1,56	2,89	-1,41	-2,67	1,14	-2,28	2,61	1,35
miR-513b-5p	1,89	1,25	-1,04	-2,98	1,12	-2,41	2,65	1,51
miR-519c-3p	1,35	1,57	-1,28	-2,39	1,18	-2,32	2,32	1,28
miR-519d-3p	1,42	1,39	-1,31	-2,58	1,11	-2,37	2,45	1,31
miR-520d-3p	1,56	1,81	-1,24	-2,78	1,02	-2,28	2,44	1,08
miR-520a	1,58	1,78	-1,44	-1,05	1,04	-2,25	2,51	1,00
miR-524-5p	1,15	1,52	-1,49	-2,99* (0.01)	3,05* (0.01)	-2,33	2,66	1,19
miR-543	1,61	1,83	-1,51	-2,85	1,21	-2,39	2,35	1,56
miR-545-3p	1,34	1,45	-1,61	-2,69	1,00	-2,37	2,13	1,59
miR-548c-3p	1,25	1,29	-1,23	-2,34	1,01	-2,65	2,29	1,71
miR-548d-3p	-1,79	-1,54	-1,13	-2,01	-2,11	-2,84	3,65	1,64
miR-548e-3p	7,06* (0.002)	5,41* (0.004)	-1,40	-2,98* (0.009)	-1,50	5,84* (0.005)	2,68	3,11
miR-590-5p	6,66* (0.003)	1,80	-1,38	-2,99* (0.01)	1,24	-4,05* (0.006)	-1,27	-2,12
miR-607	1,56	1,67	-1,41	-2,89	1,01	-2,27	2,55	1,32
miR-655-3p	1,58	1,23	-1,34	-2,78	1,09	-2,34	2,09	1,29
miR-656-3p	1,34	1,51	-1,25	-2,95	1,15	-2,25	2,78	1,42
miR-875-3p	1,45	1,20	-1,29	-2,91	1,00	-2,64	2,21	1,05

Results are expressed as fold-change compared to control values, and represent the mean value of duplicate samples from three independent experiments.

Color code: light yellow <4-fold up-regulations; yellow ≥ 4-fold up-regulations; light blue < 4-fold down-regulations; blue ≤ 4-fold down-regulations.

*, statistically significant values ($p_c \leq 0.01$).

Exact p_c values are indicated in parentheses.
